# Extended Treatment with Apixaban for Venous Thromboembolism Prevention in the Netherlands: Clinical and Economic Effects

**DOI:** 10.1055/s-0038-1672185

**Published:** 2018-09-26

**Authors:** Lisa A. de Jong, Judith J. Gout-Zwart, Jelena Stevanovic, Harrie Rila, Mike Koops, Menno V. Huisman, Maarten J. Postma

**Affiliations:** 1 Unit of PharmacoTherapy, PharmacoEpidemiology and PharmacoEconomics (PTE2), University of Groningen, Groningen, The Netherlands; 2 Department of Nephrology, University Medical Center Groningen, Groningen, The Netherlands; 3 Asc Academics, Groningen, The Netherlands; 4 Bristol Myers Squibb, Utrecht, The Netherlands; 5 Department of Thrombosis and Hemostasis, Leiden University Medical Centre (LUMC), Leiden, The Netherlands; 6 Department of Health Sciences, University of Groningen, University Medical Center Groningen (UMCG), Groningen, The Netherlands

**Keywords:** cost-effectiveness, venous thrombosis, apixaban, lifelong treatment, non–vitamin K oral anticoagulants

## Abstract

**Background** Dutch guidelines advise extended anticoagulant treatment with direct oral anticoagulants or vitamin K antagonists for patients with idiopathic venous thromboembolism (VTE) who do not have high bleeding risk.

**Objectives** The aim of this study was to analyze the economic effects of extended treatment of apixaban in the Netherlands, based on an updated and adapted previously published model.

**Methods** We performed a cost-effectiveness analysis simulating a population of 1,000 VTE patients. The base-case analysis compared extended apixaban treatment to no treatment after the first 6 months. Five additional scenarios were conducted to evaluate the effect of different bleeding risks and health care payers' perspective. The primary outcome of the model is the incremental cost-effectiveness ratio (ICER) in costs (€) per quality-adjusted life-year (QALY), with one QALY defined as 1 year in perfect health. To account for any influence of the uncertainties in the model, probabilistic and univariate sensitivity analyses were conducted. The treatment was considered cost-effective with an ICER less than €20,000/QALY, which is the most commonly used willingness-to-pay (WTP) threshold for preventive drugs in the Netherlands.

**Results** The model showed a reduction in recurrent VTE and no increase in major bleeding events for extended treatment in all scenarios. The base-case analysis showed an ICER of €9,653/QALY. The probability of being cost-effective for apixaban in the base-case was 70.0% and 91.4% at a WTP threshold of €20,000/QALY and €50,000/QALY, respectively.

**Conclusion** Extended treatment with apixaban is cost-effective for the prevention of recurrent VTE in Dutch patients.

## Introduction

Venous thromboembolism (VTE) can occur in the form of deep venous thrombosis (DVT) or pulmonary embolism (PE). In 2015, almost 86,000 patients were registered for treatment of VTE at the Dutch Thrombosis Service. 1 Patients who experienced VTE are more likely to develop recurrent VTE over time. Research has shown that in patients with idiopathic VTE, the occurrence of a recurrent VTE is 11% after the first year and up to 40% in the 10 years after the first event. 2 Currently, the standard treatment for VTE is daily treatment with direct oral anticoagulants (DOACs) for at least 3 months, or a 5- to 7-day treatment with low-molecular-weight heparin (LMWH) followed by at least a 3-month course of vitamin K antagonists (VKAs). 3 4 This advice has recently been implemented in the Dutch guidelines for general practitioners (NHG, Dutch: Nederlands Huisartsen Genootschap) as well. 5 6 The duration of treatment is determined by several factors including the exact cause of the thromboembolic episode and the patient's bleeding risk. A meta-analysis incorporated in the UK's National Institute for Health and Care Excellence (NICE) guideline shows a decrease in VTE recurrence when patients were treated for 6 months or more compared with 3 months. 7 However, it is also stated that this prolonged treatment may increase the risk of bleeding.

The thromboembolic and bleeding effects of various anticoagulation periods were also examined among different anticoagulant drugs. The Apixaban for the Initial Management of Pulmonary Embolism and Deep-Vein Thrombosis as First-Line Therapy (AMPLIFY) trial compared the efficacy and safety of the DOAC apixaban to LMWH followed by the VKA warfarin in the treatment of VTE. 8 Apixaban, given in a dose of 10 mg twice daily for 7 days followed by 5 mg twice a day for a total of 6 months, was noninferior in the prevention of recurrent VTE and statistically significantly safer than VKA. In the AMPLIFY extension (AMPLIFY-EXT) trial, after an initial course of 3 to 12 months of anticoagulant treatment patients were given either 2.5 or 5 mg apixaban twice daily or placebo for another 12 months. 9 Both doses showed a statistically significant reduction in recurrent thromboembolic events (fatal or nonfatal), while there was no statistical difference in risk of major bleeding (MB) compared with placebo.

Owing to the results of various studies, the American College of Chest Physicians (ACCP) guidelines suggest extended treatment for patients experiencing an unprovoked VTE with a low to moderate bleeding risk, 3 which is endorsed by the Dutch guidelines. 4

As stated earlier, the extended use of apixaban will result in a decrease of recurrent VTE events. Therefore, we hypothesize that treatment with apixaban in VTE patients might prevent hospitalizations and chronic complications, such as chronic thromboembolic pulmonary hypertension (CTEPH) and postthrombotic syndrome (PTS), which are associated with high costs. Moreover, prevention of these events avoids decreases in the patients' quality of life (QoL). Obviously, additional potential bleeding events, as well as the drug costs, should be taken into account when choosing the optimal treatment strategy. It is important to provide an overview of these costs and benefits, within the framework of a cost-effectiveness model. The aim of the study was to explore the clinical and economic consequences of extended treatment with apixaban versus no extended treatment in the Netherlands, based on an updated and adapted, previously published cost-effectiveness model of apixaban in VTE patients. 10

## Methods

### Cost-effectiveness Model

We performed a cost-effectiveness analysis (CEA) using a Markov model. The structure of the model was in essence the same as our previously published model used for cost-effectiveness for acute (6 months) treatment of VTE. 10 Supplementary Fig. S1 outlines the structure of the model, in which patients were able to move through 12 different health states. All patients entered the model either in the index DVT (66%) or the index PE (34%) state. 9 Since we wanted to calculate the cost-effectiveness over lifetime, we modeled a hypothetical population of 1,000 patients who were able to move among the 12 health states: recurrent VTE (recurrent PE or DVT), VTE-related death, MB, clinically relevant nonmajor bleed (CRNMB), CTEPH, death from other causes (background mortality), treatment discontinuation, or no event. In addition to the health states listed earlier, PTS is modeled in the background, so that costs and utility decrements associated with PTS were also taken into account. The model allows patients to move among the different health states with a 3-month cycle length. Transition probabilities reflect the patients' chances to move among these different health states within the cycle length. The transition probabilities for the first six cycles (18 months) were based on the AMPLIFY trials. 8 9 The consecutive cycles were based on real-world data, since the AMPLIFY trials had a follow-up time of only 18 months. 11 Subsequently, we calculated per cycle the number of patients in each health state and multiplied this with state-specific costs and health effects. Health effects are calculated in quality-adjusted life-year (QALY), with one QALY defined as 1 year in perfect health. The primary outcome of the model is the incremental cost-effectiveness ratio (ICER) in costs (€) per QALY.

**Fig. 1:**
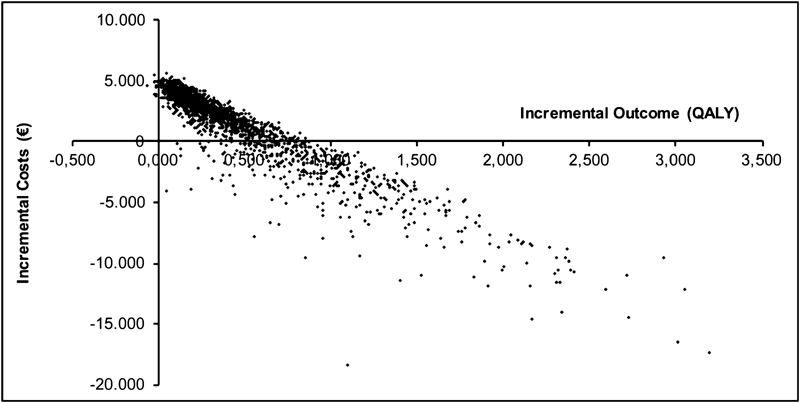
Probabilistic sensitivity analysis—base-case analysis. QALY, quality-adjusted life-years.

Patients who experienced an index VTE require anticoagulation treatment and subsequent treatment for the secondary prevention of recurrent events. Treatment duration in the model could be varied from 6 months to lifelong, in which the first 6 months of treatment was defined as the initial (or VTE treatment) period. 8 Anticoagulation treatment in the initial period was based on the AMPLIFY trial, comparing 5 mg apixaban twice daily to LMWH/VKA treatment. The extended treatment period (after 6 months of initial treatment) was considered to model the prevention of recurrent VTE by comparing continued treatment with apixaban 2.5 mg twice daily (registered dose of apixaban for this indication 12 ) to no treatment. The hypothetical model population consisted of 1,000 patients (mean age: 56.9 years, 58% male 9 ) who had an index VTE. It was assumed that 34% of the patients had experienced an initial PE and 66% had an initial DVT event, based on the patients' characteristics reported in the AMPLIFY-EXT trial. 9

Patients were followed up over lifetime with starting age of 56.9 years (maximum age: 100 years), receiving either extended treatment with apixaban or no treatment. To account for differences in MB and CRNMB between apixaban 2.5 mg and placebo in the AMPLIFY-EXT trial, additional scenario analyses were conducted. In these scenarios, the risks of MB, CRNMB, and MB plus CRNMB in the “no treatment” arm were assumed to be equal to the risks observed for apixaban. The following analyses were conducted:

- *Base-case analysis* : A CEA comparing extended treatment with apixaban to no treatment from a societal perspective. In this base-case analysis, the initial (6 months) treatment was explicitly excluded from the calculations. The efficacy and safety data of the two treatment strategies in the model were based on the AMPLIFY-EXT study (apixaban 2.5 mg vs. placebo). 9
- *Scenario analysis 1* : A CEA from a societal perspective comparing initial apixaban treatment (6 months) followed by extended treatment with apixaban to initial treatment with LMWH/VKA without extended treatment, in which efficacy and safety data were based on the AMPLIFY and AMPLIFY-EXT trial. 8 9
- *Scenario analysis 2* : The base-case analysis conducted from a healthcare payers' perspective.
- *Scenario analysis 3* : The base-case analysis where the risk of MB with no treatment is equal to the risk of MB with apixaban treatment observed in the AMPLIFY-EXT trial.
- *Scenario analysis 4* : The base-case analysis, where the risk of CRNMB with no treatment is equal to the risk of CRNMB with apixaban treatment observed in the AMPLIFY-EXT trial.
- *Scenario analysis 5* : The base-case analysis, where both MB and CRNMB risks with no treatment are equal to the risk with apixaban treatment observed in the AMPLIFY-EXT trial.

### Transition Probabilities

The transition probabilities used in the model for the extended treatment period are summarized in Table 1 . Probabilities of the initial treatment period, used in scenario 1, can be found in our previous CEA publication on the acute treatment of VTE with apixaban. 10

**Table 1:** Base-case transition probabilities used in the Markov model for the extended treatment period

Transition probability	Value (95% CI)	Distribution	Reference
Recurrent VTE and VTE-related death			
Apixaban 6–9 mo	0.0048 (0.0001–0.0094)	Beta	9
Apixaban 9–12 mo	0.0059 (0.0007–0.0111)	Beta	9
Apixaban 12–15 mo	0.0012 (0.0000–0.0035)	Beta	9
Apixaban 15–18 mo	0.0036 (0.0000–0.0076)	Beta	9
No treatment 6–9 mo	0.0277 (0.0166–0.0389)	Beta	9
No treatment 9–12 mo	0.0265 (0.0156–0.0375)	Beta	9
No treatment 12–15 mo	0.0217 (0.0118–0.0316)	Beta	9
No treatment 15–18 mo	0.0121 (0.0046–0.0195)	Beta	9
Distribution of PE, DVT, and VTE-related death			
VTE-related death	0.1188	Dirichlet	9
Recurrent PE	0.2475	Dirichlet	9
Recurrent DVT	0.6337	Dirichlet	9
Cumulative incidence of risk recurrent VTE posttreatment cessation			
0–1 y	0.0110 (0.0950–0.1250)	Beta	11
1–3 y	0.1960 (0.1750–0.2170)	Beta	11
3–5 y	0.2910 (0.2630–0.3190)	Beta	11
5–10 y	0.3990 (0.3540–0.4440)	Beta	11
MB (risk beyond first 6-mo treatment)			
Apixaban	0.0024 (0.0000–0.0057)	Beta	9
No treatment	0.0048 (0.0001–0.0096)	Beta	9
Proportion of fatal MB among MB, and nonfatal IC bleeding among nonfatal MB			
Fatal major bleeding	0.1346 (0.1128–0.1580)	Beta	13
Nonfatal IC bleeding	0.1397 (0.1160–0.1652)	Beta	13
Risk of CRNMB (risk beyond first 6-mo treatment)			
Apixaban	0.0300 (0.0182–0.0412)	Beta	9
No treatment	0.0230 (0.0128–0.0332)	Beta	9
Bleeding risk adjustment factor, major bleeding, and CRNMB (per decade)	1.970 (1.7900–2.1600)	Log normal	14
Risk of treatment interruption after non-IC bleeding (14 d)	0.4727 (0.3434–0.6039)	Beta	9
Risk of treatment interruption after CRNMB (2 d)	1.0000	Fixed	Assumption
Risk of other treatment discontinuation (unrelated to events included in the model)			
Apixaban	0.0667 (0.0498–0.0835)	Beta	9
Annual risk of CTEPH in PE patients	0.0125 (0.0003–0.0246)	Beta	15
5-y risk of severe PTS in DVT patients	0.0812 (0.0500–0.1000)	Beta	11
Hazard ratios mortality risks			
Index DVT (HR)	4.41 (3.63–5.36)	Gamma	16
Index PE (HR)	4.41 (3.63–5.36)	Gamma	16
Post-IC bleed (HR)	2.60 (2.20–5.60)	Gamma	11
Post-CTEPH (HR)	1.30 (0.98–1.73)	Gamma	17

Abbreviations: CRNMB, clinically relevant nonmajor bleeding; CTEPH, chronic thromboembolic pulmonary hypertension; DVT, deep venous thrombosis; IC, intracranial; MB, major bleeding; PE, pulmonary embolism; VTE, venous thromboembolism.

Note: Distributions were used to conduct the probabilistic sensitivity analysis.

Thromboembolic risks were estimated in a secondary analysis of the AMPLIFY-EXT trial to differentiate between different periods. The absolute risks of recurrent VTE and VTE-related death were considered to be time dependent, with risks presented for the extended treatment periods 6–9 months, 9–12 months, 12–15 months, and 15–18 months. The risk during the first 3 months (6–9 months) of extended treatment was calculated using the number of patients experiencing an event in the first 3 months as the numerator and the denominator was the intention-to-treat (ITT) population for efficacy and safety outcomes. For the subsequent intervals (9–12 months, 12–15 months, and 15–18 months), the risk of efficacy outcomes was calculated using the number of patients experiencing a recurrent event during the interval of interest as the numerator and the ITT or the modified ITT population as the denominator after censoring patients who experienced an event in any of the preceding 3-month intervals. For risks of recurrent VTE beyond 18 months, the baseline risk from Prandoni et al is used combined with the treatment effect from AMPLIFY-EXT on placebo and apixaban is applied to determine risk of patients treated with apixaban. 11

The bleeding risks for the lifelong apixaban and no treatment alternative used for extended treatment period are not time dependent due to the small number of events observed in the AMPLIFY-EXT trial. 9 For bleeds that occur in the period after 18 months, the model uses constant risks from the placebo arm of the AMPLIFY-EXT trial, adjusted with risk adjustment factors to account for aging. 11

Risks of the chronic complications of PE and DVT, such as CTEPH and PTS, were obtained from previously published articles. 2 15 Treatment discontinuation could occur due to intracranial (IC), non-IC MB, or reasons unrelated to VTE and bleeding events. It was assumed that after an IC bleed, the treatment discontinuation was permanent. 10 18 Discontinuation due to non-IC MB and CRNMB was assumed to be 14 and 2 days, respectively. These are the same assumptions as made in the previously published article on apixaban in VTE patients. 10 Treatment discontinuation unrelated to clinical events was based on the AMPLIFY-EXT trial. 9 General mortality rates were based on the Dutch life tables from the Statistic Netherlands. 19

### Costs and Utilities

Cost and utility parameters were the same as presented in the previous publication of the acute treatment model ( Supplementary Tables S1 and S2 ). 10 The primary economic outcome of the model was the ICER, calculated by dividing the difference in costs by the difference in QALYs. In the base-case analysis, the ICER was calculated from a societal perspective, as recommended in the Dutch guidelines for cost-effectiveness studies. 20 Therefore, all relevant costs were taken into account including indirect costs, such as productivity losses. Scenario 2 was specifically calculated from a healthcare payers' perspective, excluding these indirect costs. Annual discount rates for costs and utilities of 4 and 1.5% were used, respectively. 20 All costs were updated to the year 2015.

**Table 2:** Recurrent VTE, bleeding events, and other adverse events and corresponding costs within a hypothetical cohort of 1,000 VTE patients: the base-case results

	Extended apixaban		No treatment	
	Events, *n*	Cost/Patient	Events, *n*	Cost/Patient
Recurrent VTE and VTE-related death				
VTE-related death	35	€13	72	€31
Nonfatal recurrent PE	72	€109	149	€250
Nonfatal recurrent DVT	185	€34	382	€79
* Total*	*292*	*€156*	*603*	*€360*
Major bleeds				
Fatal	14	€177	18	€233
Nonfatal intracranial bleed	13	€232	16	€313
Nonfatal extracranial bleed	80	€241	100	€316
* Total*	*107*	*€650*	*134*	*€862*
Clinically relevant nonmajor bleeds	769	€14	641	€12
CTEPH	31	€254	33	€269
Treatment discontinuation	585		50	
Anticoagulant and administration costs		€5,686		€84
Monitoring costs		€12		€39
Indirect costs				
Productivity loss		€2,241		€3,847
Transportation costs		€95		€227

Abbreviations: CTEPH, chronic thromboembolic pulmonary hypertension; DVT, deep venous thrombosis; PE, pulmonary embolism; VTE, venous thromboembolism.

In the extended treatment period, drug costs were based on 2.5 mg apixaban twice daily. In scenario 1, we included the initial treatment period, which was specified as 7 days 10 mg followed by 6 months 5 mg apixaban twice daily. This was compared with a 5-day LMWH treatment (171 anti-Xa IU/kg/day) and 7 days of 3.2 mg VKA (acenocoumarol, phenprocoumon) treatment daily followed by 2.2 mg VKA daily. Drug costs were obtained from the official Dutch price list (Z-index) and were assigned to all the health states excluding patients in their treatment discontinuation period as described earlier. 21

Costs of PE and DVT for in- and outpatients were based on the declaration costs published by the Dutch Healthcare Authority. 22 Other event costs, such as CTEPH, PTS, and bleeding, are based on various previously published articles. 23 24 25 26 Direct costs outside the healthcare sector referred to travel expenses. Travel expenses were taken into account at the occurrence of a hospital administration or visit, international normalized ratio (INR) monitoring visit or a hospital visit for IC bleed, CTEPH, PE, or DVT event. The tariff was based on a mean of a one-way 7-km traveling distance by car, public transportation, and taxi, as described in the Dutch guidelines for pharmaco-economic research. 20 Indirect costs outside the healthcare sector referred to productivity losses, calculated with the friction cost method. 20 Hourly wages and the probability of employment were based on information from the Statistics Netherlands. 19 All costs parameters are summarized in Supplementary Table S1 .

The health effects in the model were calculated in QALYs, reflecting life-years adjusted for their utility. The baseline utility for patients in the model referred to the average for 60- to 70-year-old Dutch men and women estimated with the five-level version of the EQ-5D. 27 Upon the (re-)occurrence of VTE or bleeding events, utility decrements were applied to values specific for each health state. Utility parameters are presented in Supplementary Table S2 .

### Sensitivity Analysis

To account for influence of the uncertainties on the economic outcomes of the model, sensitivity analyses were conducted. In the probabilistic sensitivity analysis (PSA), all uncertain parameters were simultaneously varied over their 95% confidence interval (CI), while calculating the ICER 2,000 times. For event risks, multipliers, and distributions of the risks, we used β, log normal, and Dirichlet distributions, respectively. We used a gamma or log normal distribution for costs and β distributions for utilities. 28 All 2,000 iterations were plotted in a cost-effectiveness plane. Subsequently, a cost-effectiveness acceptability curve (CEAC) was obtained to show the probability of being cost-effective at different willingness-to-pay (WTP) thresholds. The probability of being cost-effective based on the iterations was subsequently calculated in the PSA.

In the univariate sensitivity analysis (USA), we varied all relevant parameters individually over their 95% CI to explore which parameters had the highest influence on the ICER. When the 95% CI was not available, a standard error of 30% was used. 28

### Model Validation

Predictive validity was checked by comparing key outcomes to the source data. In performing this validation, the model was set to the settings of the AMPLIFY-EXT and the AMPLIFY (i.e., number of patients, duration of follow-up) and the key event rates were compared.

## Results

The clinical results of the base-case and scenario analyses are shown in Table 2 and Supplementary Table S3 , respectively. In the base-case cohort of 1,000 VTE patients in total, 310 recurrent VTE, 27 MBs, and 2 cases of CTEPH are prevented with extended treatment compared with no treatment, based on efficacy and safety data from the AMPLIFY-EXT trial. Extended apixaban treatment increases the number of CRNMBs and treatment discontinuation, compared with no treatment.

**Table 3:** Costs and effects per patient and incremental cost-effectiveness ratios in the base-case and scenario analyses

	Extended apixaban	No treatment	Incremental
Base-case: Extended treatment with apixaban vs. no treatment based on AMPLIFY-EXT (societal perspective)			
Total costs	€10,110	€6,643	€3,468
Total QALYs	10.971	10.612	0.359
Cost per QALY gained			€9,653
Scenario 1: Initial + extended treatment with apixaban vs. initial treatment with LMWH/VKA followed by no treatment (societal perspective)			
Total costs	€11,203	€8,229	€2,974
Total QALYs	10.908	10.540	0.368
Cost per QALY gained			€8,085
Scenario 2: Base-case analysis from a healthcare payers' perspective			
Total costs	€7,775	€2,570	€5,205
Total QALYs	10.971	10.612	0.359
Cost per QALY gained			€14,490
Scenario 3: Base-case analysis where the MB risk is equal for apixaban and no treatment			
Total costs	€9,768	€5,921	€3,847
Total QALYs	11.007	10,692	0.314
Cost per QALY gained			€12,267
Scenario 4: Base-case analysis where the CRNMB risk is equal for apixaban and no treatment			
Total costs	€10,112	€6,647	€3,466
Total QALYs	10.971	10.612	0.359
Cost per QALY gained			€ 9,648
Scenario 5: Base-case analysis where the MB and CRNMB risks are equal for apixaban and no treatment			
Total costs	€9,770	€5,924	€3,845
Total QALYs	11.007	10.692	0.314
Cost per QALY gained			€12,231

Abbreviations: CRNMB, clinically relevant nonmajor bleeding; LMWH, Low-molecular-weight heparin; MB, major bleeding; QALY, quality-adjusted life-year; VKA, vitamin K antagonist.

The deterministic economic results are shown in Table 3 . In the base-case scenario, lifelong apixaban treatment shows incremental costs of €9,653 per QALY compared with no treatment. The first scenario shows an ICER of €8,085 per QALY for initial apixaban treatment followed by extended treatment, compared with initial LMWH/VKA treatment followed by no treatment. The base-case calculated from a healthcare payers' perspective (scenario 2) shows an ICER of €14,490 per QALY. Three additional scenarios (scenarios 3–5) were conducted to explore the effect of equal bleeding risks resulting in ICERs of €12,267, €9,648, and €12,231 per QALY, respectively.

Table 2 shows the number of events and the corresponding costs per patient in the hypothetical population of 1,000 VTE patients. The costs per patient per event reflect the number of events that occurred in the hypothetical population multiplied by the costs of the event divided over the whole population. MBs and productivity losses showed to have a big contribution on total costs. In the apixaban arm, the highest costs per patient were spent on drug costs. Similar results were found for the scenarios ( Supplementary Table S3 ).

**Fig. 2:**
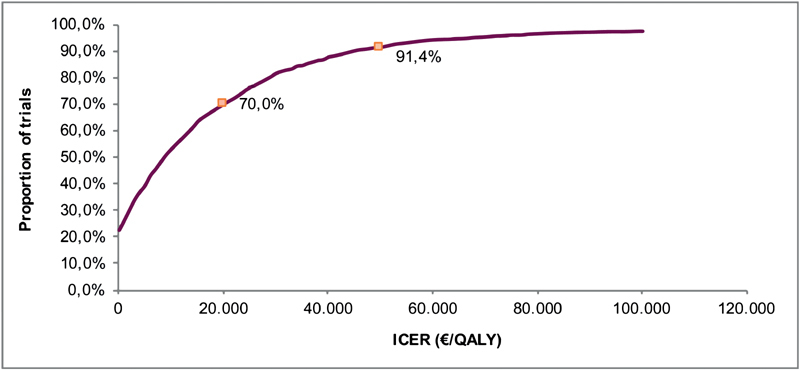
The cost-effectiveness acceptability curve (CEAC)—base-case analysis. With the probabilities of being cost-effective at a WTP threshold of €20,000/QALY and €50,000/QALY. ICER, incremental cost-effectiveness ratio; QALY, quality-adjusted life-year.

**Fig. 3:**
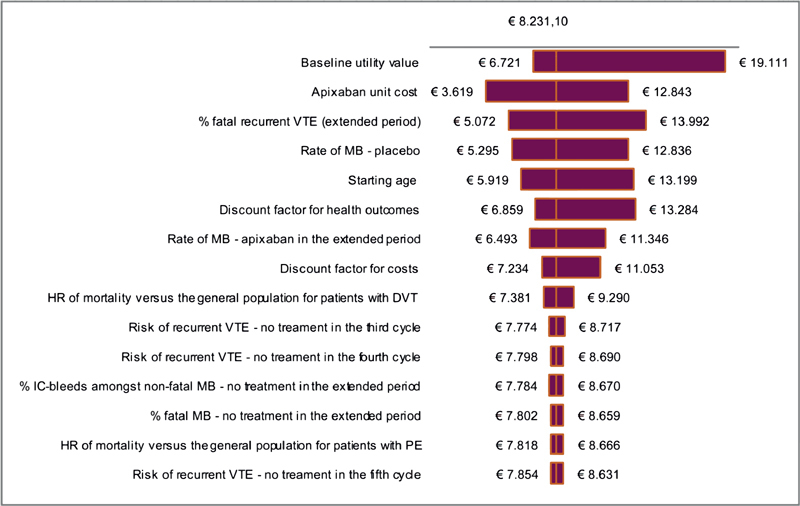
Tornado diagram resulting from the univariate sensitivity analysis. DVT, deep venous thrombosis; HR, hazard ratio; IC, intracranial; MB, major bleeding; PE, pulmonary embolism; VTE, venous thromboembolism.

The PSA for the base-case analysis was performed with 2,000 iterations of ICER calculation, which is displayed in Fig. 1 . The corresponding CEAC of the base-case analysis is shown in Fig. 2 . The probability of being cost-effective for apixaban in the base-case was 70.0 and 91.4% at a WTP threshold of €20,000/QALY and €50,000/QALY, respectively. Fig. 3 represents the results of the USA. The baseline utility value has the highest influence on the ICER (€6,721–€19,111), followed by the apixaban unit cost (€3,619–€12,843) and the rate of MBs in patients with no extended treatment (€5,295–€12,836).

The results from the predictive validation against the AMPLIFY and the AMPLIFY-EXT trials are shown in Tables 4 and 5 , respectively. All relative risks (RRs) calculated in the model are within the 95% CI reported in the AMPLIFY and AMPLIFY-EXT trials. 8 9

**Table 4:** Model validation comparing the results of the model to the AMPLIFY trial outcomes

	AMPLIFY			Model results		
	Apixaban	LMWH/VKA	RR	Apixaban	LMWH/VKA	RR
Recurrent VTE and VTE-related death	59	71	0.83	60	72	0.83
Major bleeding	15	49	0.31	15	49	0.31
CRNMB	103	215	0.48	102	214	0.48
Treatment discontinuation	162	199	0.82	131	150	0.87
All-cause death	41	52	0.79	40	47	0.85

Abbreviations: CRNMB, clinically relevant nonmajor bleeding; LMWH, low-molecular-weight heparin; RR, relative risk; VKA, vitamin K antagonist; VTE, venous thromboembolism.

Note: Model settings—number of patients in each treatment group: apixaban, *N* = 2691; LMWH/VKA, *N* = 2704; treatment duration and time horizon = 6 months.

**Table 5:** Model validation comparing the results of the model to the AMPLIFY-EXT trial outcomes

	AMPLIFY-EXT			Model results		
	Apixaban	Placebo	RR	Apixaban	Placebo	RR
Recurrent VTE and VTE-related death	14	73	0.19	13	72	0.18
Major bleeding	2	4	0.49	2	4	0.50
CRNMB	25	19	1.29	24	19	1.26
All-cause death	32	96	0.33	31	97	0.32

Abbreviations: CRNMB, clinically relevant nonmajor bleeding; RR, relative risk; VTE, venous thromboembolism.

Notes: Model settings—number of patients in each treatment group: apixaban 2.5 mg, *N* = 804; LMWH/VKA, *N* = 829; treatment duration and time horizon = 12 months.

## Discussion

This CEA provides insight in the economic effects of extended treatment with apixaban. Although exact numbers of chronic users of apixaban or VKA as prophylaxis for VTE events in the Netherlands is unknown, 1 guidelines are very clear: continued treatment is essential in case of recurrent idiopathic VTE. 4 Therefore, it is important to estimate the economic effects of this treatment, especially since previously published articles on the CEA of apixaban focused only on the short-term (6 up to 18 months) treatment. 29

In the base-case analysis, extended treatment with apixaban versus no treatment can be considered to be cost-effective with an ICER of €9,653/QALY when compared with the minimal WTP threshold of €20,000/QALY, commonly used in the Netherlands (notably, also higher WTPs are sometimes mentioned, such as €50,000/QALY). 30 The PSA found that apixaban in the base-case had a probability of being cost-effective of 70.0 and 91.4% at a WTP threshold of €20,000/QALY and €50,000/QALY, respectively. The scenario analysis including the initial 6-month treatment as well as the preventive extended treatment period (scenario 1) turned out to be even more cost-effective. Scenario 2, in which we calculated the ICER from healthcare payers' perspective, was performed to make the results of the CEA comparable to other countries who use this perspective, like for example the United Kingdom. With an ICER of €14,490/QALY, this scenario was cost-effective.

Contrary to potential expectations, the number of patients in the AMPLIFY-EXT experiencing a MB was two versus four in the apixaban 2.5 mg and placebo group, respectively. The number of patients experiencing a CRNMB was 25 for apixaban 2.5 mg and 19 in the placebo group. 9 Therefore, we explored additional scenarios that assume equivalent bleeding risks (separately for MBs and CRNMBs) for both treatment arms. Results of these scenarios still indicate apixaban lifelong treatment to be cost-effective compared with no treatment, which can be explained by the fact that these were not statistically significant differences and would therefore not warrant such an important effect on costs if taken as starting point for lifelong calculations. Supplementary Table S3 shows that, despite the fact that the bleeding risks are assumed equal, small differences in bleeding numbers were still observed. This can be explained by the fact that risks of all other events did differ, causing different numbers of patients to be at risk of bleeding, resulting in slightly different outcomes.

Notably, the costs per patient for bleeding events are higher compared with costs per patient related to VTE. Bleedings, especially IC bleeds and MB, are related to very high expenses. Despite the fact that anticoagulation therapy might increase costs per patient, preventing either VTE or bleeding events is beneficial for the patient's health and therefore these increasing costs are inferior to the overall increase in QALYs. Another striking point in Table 2 is the monitoring costs in the “no treatment” group. This can be explained by the fact that patients who were not on treatment were nevertheless at risk of recurrent VTE. All patients who experienced a recurrent VTE in the “‘no treatment” group were assumed to resume LMWH/VKA treatment since they were treated with this same treatment during the initial treatment period (first 6 months), based on the AMPLIFY and AMPLIFY-EXT trial. 8 9 Monitoring costs of apixaban are low compared with VKA. Therefore, the “no treatment” group still had higher monitoring costs than the apixaban-treated group over lifetime.

In the validation of the model ( Tables 4 and 5 ), most of the RRs were comparable to the outcomes reported from the trials, apart from all-cause mortality and treatment discontinuation. The differences observed in death rates can be attributed to the use of specific Dutch background mortality rates. The lower number of discontinuations observed can be attributed to patients discontinuing treatment upon the occurrence of VTE or CRNMBs in the trial, whereas in our model we allowed for these patients to remain on treatment.

Various studies already showed apixaban being cost-effective or even cost-saving compared with LMWH/VKA in Dutch VTE and atrial fibrillation patients. 10 25 31 To our knowledge, this is the first CEA on extended treatment with apixaban for VTE prevention in the Netherlands. Long-term apixaban cost-effectiveness studies have already been conducted in the United Kingdom and Spain. Elìas et al compared lifelong apixaban treatment with lifelong LMWH/VKA treatment, based on the AMPLIFY and AMPLIFY-EXT study and calculated an ICER of €4,751/QALY. 32 Lanitis et al reported an ICER of £16,944/QALY for this same scenario. 29 More comparable to our study is the scenario conducted by Lanitis et al in which initial LMWH/VKA followed by no treatment was compared with extended apixaban treatment and resulted in an ICER of £13,107/QALY. This result seems comparable to the results of this study. Small differences in outcomes are due to differences in costs, perspectives, utilities, and background mortalities across countries.

As with all models, there are limitations to our analysis. This study utilized a Markov approach, which is limited by the memory-less assumption. As a result, we did not model any further recurrent VTE events after IC bleeds, nor did we account for an increased risk of additional recurrent VTE once patients experienced a recurrent event. For the analysis, we had to rely on trial data and real-world data of 528 symptomatic DVT patients combined with AMPLIFY trial data. 11 Trials are often done in controlled setting which does not reflect the real-world clinical setting. In future research, long-term use of apixaban should be followed to determine the real-life effectiveness.

To make the results of the CEA of lifelong treatment comparable to short-term treatment, costs were, similar to the previously published publication, inflated to the year 2015 according to Dutch Statistics. The inflation numbers in 2016 and 2017 were relatively stable with 0.3 and 1.4%, respectively. 33 The assumptions made in our model are likely to be unfavorable to apixaban given the higher recurrent VTE reduction rates seen with apixaban treatment. We assumed that the origin of recurrent VTE and MB events was independent of treatment. This assumption is similar to that used in other models 34 35 and is appropriate given that the trials were not sufficiently powered to determine differences in the individual end-points. Beyond the 6 months of initial treatment period, data from AMPLIFY-EXT were used to model the risk of recurrent VTE for the next 12 months. Real-world data were used to model the risk thereafter. 11 Results might differ between trial and real-world studies; therefore, all thromboembolic and bleeding risks were tested in the probabilistic as well as the USA which showed to be robust.

Several sources of utility and cost values were available for the health-states modeled. In our analysis, we used Dutch tariffs where possible, but these were not available for all health states. The USA shows results are most sensitive to the baseline utility value and apixaban unit cost; however, varying these parameters the ICER remains below the €20,000/QALY threshold. Therefore, motivations for both under- and overestimation of cost-effectiveness results exist, but sensitivity analyses examining these uncertainties did not alter the conclusions of our analysis.

We conclude that continued apixaban treatment is a cost-effective approach for the treatment and prevention of (recurrent) VTE. Moreover, results of the probabilistic, univariate, and scenario analyses seem very robust. These health economic results underline the current guideline recommendation for extended anticoagulant treatment with apixaban. 3 4
