# Supplementary material for: Extended Treatment with Apixaban for Venous Thromboembolism Prevention in the Netherlands: Clinical and Economic Effects
Source: TH Open. 2018 Sep 26;2(3):e315–24. doi: 10.1055/s-0038-1672185 (PMC6524888; doi:10.1055/s-0038-1672185)
Supplement: Supplementary file 1 — Supplementary Material [file 10-1055-s-0038-1672185-s180006.pdf]

Supplementary Material

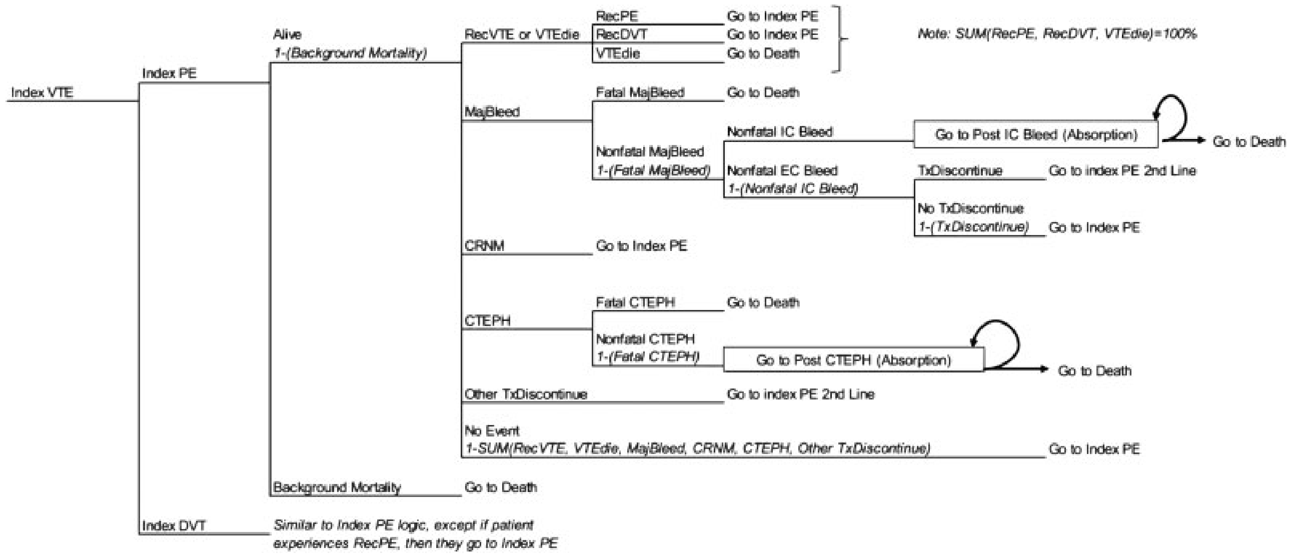

Supplementary Fig. S1 Schematic structure of the cohort-based Markov model for VTE patients.[1] CRNMB, clinically relevant nonmajor bleed; CTEPH, chronic thromboembolic pulmonary hypertension; DVT, deep venous thromboembolism; EC, extracranial; IC, intracranial; MB, major bleeding; PE, pulmonary embolism; Rec, recurrent; TxDiscontinue, treatment discontinuation; VTEdie, venous thromboembolism-related death.

Supplementary Table S1 Costs parameters used in the Markov model

Resources	Average cost (€, 2015)	Range (€)	Reference
Medication			
Apixaban (daily)			
Introduction period (20 mg/d)	4.60	Fixed	[2]
Long-term period (10 mg/d)	2.30	Fixed	[2]
Extended treatment (2.5 mg/d)	2.15	Fixed	[2]
LMWH: nadroparin 19,000 IU/mL, 0.8 mL (daily)	9.37	Fixed	[2]
VKA (daily)			
Introduction period (3.2 mg)	0.06	Fixed	[2]
Long-term period (2.2 mg)	0.04	Fixed	[2]
Administration and monitoring costs			
Patient education/instruction to self-inject	71.32	53.49–89.15	[3]
LMWH administration by homecare nurse	19.05	14.29–23.81	[3]
INR monitoring visit	11.58	8.69–14.48	[4]
Event-related costs			
PE inpatients	2,150.60	1,229–3,326	[3]
DVT inpatients	604.89	345.95–935.44	[3]
PE/DVT outpatients			
Doppler ultrasound	41.09	30.82–51.36	[3]
CT angiography	204.25	153.19–255.31	[3]
Echocardiogram	41.09	30.82–51.36	[3]
D-Dimer test	2.45	1.84–3.06	[3]
GP visit	29.20	21.90–36.50	[3]
Long-term maintenance	253.01	126.51–379.53	[5]
Non-IC major bleeding	5,036	3,664–6,921	[6]
CRNMB (one GP visit)	30.72	23.04–38.40	[6]
Severe PTS	447.24	313.07–581.41	[6]
Fatal major bleeding	20,489	14,907–28,161	Assumption: costs are equal to acute care costs
Fatal event due to CTEPH	4,271	2,136–6,407	Assumption: costs are equal to acute care costs
VTE-related death	566.23	396.36–736.10	Assumption: costs are equal to acute care costs
Direct costs outside health care			
Travel expenses due to administration/monitoring visit/hospital visit for IC bleed, CTEPH, PE, or DVT event	14.76	3.09–34.78	[7]
Indirect costs outside health care			
Expected hourly wages			
Male	41.29	Fixed	[7]
Female	30.60	Fixed	[7]
Probability of employment (based on age 55–65 y)			
Male	71.0%	Fixed	[7,8]
Female	52.4%	Fixed	[7,8]

Abbreviations: CRNMB, clinically relevant nonmajor bleeding; CT, computed tomography; CTEPH, chronic thromboembolic pulmonary hypertension; DVT, deep venous thrombosis; GP, general practitioner; IC, intracranial; INR, international normalized ratio; LMWH, low-molecular-weight heparin; PE, pulmonary embolism; PTS, postthrombotic syndrome; VKA, vitamin K antagonist; VTE, venous thromboembolism.
Source: Reprinted with permission from de Jong et al.[1]

Supplementary Table S2 Utility parameters used in the Markov model

Utilities	Value	Reference	Duration	Reference
Baseline utility	0.825	9		
Post-IC bleeding state	0.330	10		
Post-CTEPH state	0.650	11		
PE	−0.32	10	30 d	12,13
DVT	−0.11	10	30 d	12,13
Non-IC major bleeding	−0.30	10	30 d	12,13
CRNMB	−0.0054	14	2 d	Assumption
Severe PTS	−0.070	12	Throughout	15
Anticoagulation				
Apixaban	−0.002	13	While on treatment	16
LMWH/VKA	−0.013	13	While on treatment	16

Abbreviations: CRNMB, clinically relevant nonmajor bleeding; CTEPH, chronic thromboembolic pulmonary hypertension; DVT, deep venous thrombosis; IC, intracranial; LMWH, low-molecular-weight heparin; PE, pulmonary embolism; PTS, postthrombotic syndrome; VKA, vitamin K antagonist.
Source: Reprinted with permission from de Jong et al.[1]

Supplementary Table S3 Recurrent VTE, bleeding events, and other adverse events and corresponding costs within a hypothetical cohort of 1,000 VTE patients: results of scenarios 1–5

	Scenario 1				Scenario 2			
	Apixaban		No treatment/VKA		Apixaban			No treatment
	Events, *n*	Costs/patient	Events, *n*	Costs/patient	Events, *n*	Costs/patient	Events, *n*	Costs/patient
**Recurrent VTE and VTE-related death**								
VTE-related death	40	€16	75	€33	35	€13	72	€31
Nonfatal recurrent PE	81	€128	154	€259	72	€109	149	€250
Nonfatal recurrent DVT	196	€37	378	€78	185	€34	382	€78
Total	*317*	*€181*	*606*	*€369*	*292*	*€156*	*603*	*€359*
**Major bleeds**								
Fatal	15	€184	19	€267	14	€177	18	€233
Nonfatal intracranial bleed	13	€245	18	€373	13	€232	16	€313
Nonfatal extracranial bleed	80	€250	108	€363	80	€241	100	€316
Total	*108*	*€679*	*145*	*€1,004*	*107*	*€650*	*134*	*€862*
CRNMB	762	€14	684	€13	769	€14	641	€12
CTEPH	32	€257	33	€269	31	€254	33	€269
Treatment discontinuation	588		102		585		50	
Anticoagulant and administration costs		€5,582		€279		€5,686		€84
Monitoring costs		€12		€176		€12		€39
**Indirect costs**								
Productivity loss		€2,450		€4,029		€0		€0
Transportation costs		€107		€227		€0		€0

(*Continued*)

Supplementary Table S3 (*Continued*)

	Scenario 3				Scenario 4			
	Apixaban		No treatment		Apixaban		No treatment	
	Events, *n*	Costs/patient	Events, *n*	Costs/patient	Events, *n*	Costs/patient	Events, *n*	Costs/patient
**Recurrent VTE and VTE-related death**								
VTE-related death	35	€14	72	€31	35	€13	72	€31
Nonfatal recurrent PE	73	€109	150	€251	72	€109	149	€250
Nonfatal recurrent DVT	186	€34	385	€79	185	€34	382	€80
Total	*294*	*€157*	*607*	*€362*	*292*	*€156*	*603*	*€359*
**Major bleeds**								
Fatal	10	€122	9	€118	14	€177	18	€233
Nonfatal intracranial bleed	9	€164	8	€152	13	€232	16	€313
Nonfatal extracranial bleed	53	€166	51	€160	80	€241	100	€316
Total	*71*	*€451*	*68*	*€436*	*107*	*€650*	*134*	*€862*
CRNMB	774	€14	651	€12	876	€16	836	€15
CTEPH	31	€255	34	€271	31	€254	33	€269
Treatment discontinuation	585		26		585		50	
Anticoagulant and administration costs		€5686		€85		€5686		€84
Monitoring costs		€12		€39		€12		€39
**Indirect costs**								
Productivity loss		€2,093		€3,542		€2,241		€3,847
Transportation costs		€95		€226		€95		€227

	Scenario 5			
	Apixaban		LMWH/VKA	
	Events, *n*	Costs/patient	Events, *n*	Costs/patient
**Recurrent VTE and VTE-related death**				
VTE-related death	35	€14	72	€31
Nonfatal recurrent PE	73	€176	150	€405
Nonfatal recurrent DVT	186	€35	385	€80
Total	*294*	*€224*	*607*	*€513*
**Major bleeds**				
Fatal	10	€122	9	€118
Nonfatal intracranial bleed	9	€164	8	€158
Nonfatal extracranial bleed	53	€166	51	€160
Total	*71*	*€451*	*68*	*€436*
CRNMB	882	€16	848	€16
CTEPH	31	€255	34	€271
Treatment discontinuation	585		26	
Anticoagulant and administration costs		€5686		€84
Monitoring costs		€12		€39
**Indirect costs**				
Productivity loss		€2,093		€3,542
Transportation costs		€95		€226

Abbreviations: CRNMB, clinically relevant nonmajor bleeding; CTEPH, chronic thromboembolic pulmonary hypertension; DVT, deep venous thrombosis; EC, extracranial; IC, intracranial; LMWH, low-molecular-weight heparin; PE, pulmonary embolism; VKA, vitamin K antagonists; VTE, venous thromboembolism.

References

1 de Jong LA, Dvortsin E, Janssen KJ, Postma MJ. Cost-effectiveness analysis for apixaban in the acute treatment and prevention of venous thromboembolism in the Netherlands. Clin Ther 2017;39(02):288–302.e4

2 The National Health Care Institute Netherlands (ZIN). [cited December 1, 2016]. Available at: www.medicijnkosten.nl

3 Dutch Healthcare Authority (NZa). Tarieven & Prestaties: Tarieventable DBC-zorgproducten en overige producten 2012. [cited December 1, 2016]. Available at: https://puc.overheid.nl/nza/zoeken/result aat/s/2/p/1/gdlv/1/g/1/srt/tarieven_en_prestaties/. Accessed August 30, 2018

4 Trombosediensten N, Toekomst I, Actief V. Annual Medical Report Dutch Federation of Thrombosis Services 2014. 2014. Available at: https://s3.eu-central-1.amazonaws.com/storage.topsite.nl/fnt.nl/uploads/docs/jaarverslagen/Jaarverslag_definitief_2014.pdf. Accessed August 30, 2018

5 Stevanović J, Pompen M, Le HH, Rozenbaum MH, Tieleman RG, Postma MJ. Economic evaluation of apixaban for the prevention of stroke in non-valvular atrial fibrillation in the Netherlands. PLoS One 2014;9(08):e103974

6 Ten Cate-Hoek AJ, Toll DB, Büller HR, et al. Cost-effectiveness of ruling out deep venous thrombosis in primary care versus care as usual. J Thromb Haemost 2009;7(12):2042–2049

7 Hakkaart-van Roijen L, van der Linden N, Bouwmans C, Kanters T, Swan Tan S. Kostenhandleiding: Methodologie van kostenonderzoek en referentieprijzen voor economische evaluaties in de gezondheidszorg. Published by: Zorginstituut Ned. 2016;1–120. Available at: www.zorginstituutnederland.nl/publicaties/publicatie/2016/02/29/richtlijn-voor-het-uitvoeren-van-economische-evaluaties-in-de-gezondheidszorg

8 Agnelli G, Buller HR, Cohen A, et al; AMPLIFY Investigators. Oral apixaban for the treatment of acute venous thromboembolism. N Engl J Med 2013;369(09):799–808

9 M Versteegh M, M Vermeulen K, M A A Evers S, de Wit GA, Prenger R, A Stolk E. Dutch tariff for the five-level version of EQ-5D. Value Health 2016;19(04):343–352

10 Locadia M, Bossuyt PMM, Stalmeier PFM, et al. Treatment of venous thromboembolism with vitamin K antagonists: patients' health state valuations and treatment preferences. Thromb Haemost 2004;92(06):1336–1341

11 Ghofrani H-A, D'Armini AM, Grimminger F, et al; CHEST-1 Study Group. Riociguat for the treatment of chronic thromboembolic pulmonary hypertension. N Engl J Med 2013;369(04):319–329

12 National Institute for Health and Care Excellence (NICE). Venous thromboembolism: reducing the risk for patients in hospital. Guidance and guidelines. 2010 [cited March 28, 2018]. Available at: https://www.nice.org.uk/guidance/cg92. Accessed August 30, 2018

13 Hogg K, Kimpton M, Carrier M, Coyle D, Forgie M, Wells P. Estimating quality of life in acute venous thrombosis. JAMA Intern Med 2013;173(12):1067–1072

14 Sullivan PW, Slejko JF, Sculpher MJ, Ghushchyan V. Catalogue of EQ-5D scores for the United Kingdom. Med Decis Making 2011;31(06):800–804

15 Lenert LA, Soetikno RM. Automated computer interviews to elicit utilities: potential applications in the treatment of deep venous thrombosis. J Am Med Inform Assoc 1997;4(01):49–56

16 Gage BF, Cardinalli AB, Owens DK. The effect of stroke and stroke prophylaxis with aspirin or warfarin on quality of life. Arch Intern Med 1996;156(16):1829–1836
